# Common Mutations of the Methylenetetrahydrofolate Reductase (MTHFR) Gene in Non-Syndromic Cleft Lips and Palates Children in North-West of Iran

**Published:** 2015-01

**Authors:** Shahin Abdollahi-Fakhim, Mehrdad Asghari Estiar, Parizad Varghaei, Mahdi Alizadeh Sharafi, Masoud Sakhinia, Ebrahim Sakhinia

**Affiliations:** 1 *Department of Pediatric Otorhinolaryngology, Children’s Hospital, Tabriz University of Medical Sciences, Tabriz, Iran. *; 2 *Department of Medical Genetics, School of Medicine, Tehran University of Medical Sciences, Tehran, Iran.*; 3 *Faculty of Medicine, Tabriz University of Medical Sciences, Tabriz, Iran.*; 4 *Tabriz Genetic Analysis Center (TGAC), Tabriz University of Medical Sciences, Tabriz, Iran.*; 5 *Faculty of Medicine, University of Liverpool, Liverpool, United Kingdom. *; 6 *Tuberculosis and Lung Disease Research Center, Tabriz University of Medical Sciences, Tabriz, Iran.*

**Keywords:** A1298C mutation, Cleft lip, Cleft palate, C677T mutation, MTHFR

## Abstract

**Introduction:**

Cleft lips and cleft palates are common congenital abnormalities in children. Various chromosomal loci have been suggested to be responsible the development of these abnormalities. The present study was carried out to investigate the association between the suspected genes (methylenetetrahydrofolate reductase [MTHFR] A1298C and C677T) that might contribute into the etiology of these disorders through application of molecular methods.

**Materials and Methods:**

This cross-sectional and explanatory study was carried out on a study population of 65 affected children, 130 respective parents and 50 healthy individuals between 2009 and 2012 at Tabriz University of Medical Sciences, IR Iran. After DNA extraction, amplification refractory mutation system–polymerase chain reaction (ARMS-PCR) and restriction fragment length polymorphism (RFLP)-PCR were used respectively to investigate the C677T and A1298C mutations for the MTHFR gene.

**Results:**

There was a significant difference in the rates of the C677T mutation when affected patients and their fathers were compared with the control group (odds ratio [OR]=0.44) (OR=0.64). However, there was no significant difference observed in the rate of this mutation between the patients’ mothers and the control group (OR=1.35). In addition, the abnormality rate was higher in patients with the A1298C mutation and their parents, when compared with the control group. This abnormality rate was higher for the affected children and their fathers in comparison with their mothers (Fathers, OR=0.26; Mothers, OR=0.65; Children, OR=0.55). No significant difference was seen in the rate of the polymorphism C677T in its CC, when the affected children and their parents were compared with the control group. However, there was a significant difference in the A1298C mutation.

**Conclusion:**

An association was seen between the A1298C mutation and cleft lip and cleft palate abnormalities in Iran. However, there seems to be a stronger relationship between the C67TT mutation and these abnormalities in other countries, which could be explained by racial differences. Moreover, this association was more notable between the affected children and their fathers than their mothers. The findings in this study may be helpful in future studies and screening programs.

## Introduction

Cleft lips and cleft palates are considered to be among the most common congenital anomalies in children. A syndromic cleft palate is usually associated with other anomalies in different organs, whilst this is not frequently observed in the non-syndromic subtype. Approximately 70 percent of cleft lips and cleft palates are thought to be non-syndromic (1,2).

Non-syndromic cleft lip and cleft palate are known as craniocephalic anomalies and require lifelong care and management. These abnormalities can cause problems in eating, speaking and hearing in the affected children. In addition, the abnormalities bring financial, social and psychological costs upon families and the wider society (3). 

The incidence and presentation of these congenital disorders can sometimes vary according to differing geographical, economic, and racial factors (3,4). In Asian and American ethnicities, the prevalence of the disorder can be as high as one case among 500 births, while the prevalence in individuals of African ethnicity is relatively lower at one in 2,500 births (5). The lowest rate belongs to the black people (3), while the highest rate is attributed to Japanese citizens (6,7). Two studies in Tehran have shown a rate of between 1.3 to 3.73 in 1,000 births in Iran (8,9). These studies suggest that the disorders carry a multi-factorial etiology in which environmental factors act on a genetically predisposed individual. Smoking, alcohol consumption during pregnancy, pregnancy-associated infections, vitamin deficiencies, and the use of some teratogenic drugs are reported to be critical factors (10-12). 

Anderson highlights the impact of genetic factors on the development of cleft palates (13,14). Approximately 20 percent of patients in various societies have a positive family history of these abnormalities (15). Furthermore, it has been shown that the ratio between males and females for cleft palates is 1.98–2.07 to 1 (4). In twins, the association of the abnormality in monozygotic twins is estimated to be around 40–60 percent, compared with only 5 percent in dizygotic twins (3,4). Accordingly, cleft lips and cleft palates are genetically non-uniform, in which the chromosomal regions have several loci responsible for their etiology (1). 

One of the most influential genetic factors in the etiology of cleft lips and cleft palates seems to be the methylenetetrahydrofolate reductase (MTHFR) gene mutation. Furthermore, the annealing between dihydrofolate and S-adenosyl methionine can halter its activity (16). The location of the MTHFR gene or coding of the MTHFR enzyme is on chromosome 1 and location p36.3 in the human genome. The sequence of DNA inside this gene is constructively variable (polymorphism). 

Since 2000, more than 24 polymorphisms have been reported as being linked to this abnormality. The most prevalent are two polymorphisms called C677T and A1298C (17). In fact, the two most common mutations of this gene result in a reduction of its activity of 20–70 percent, leading to an accumulation of homocysteine and a reduction of serum. The simultaneous presence of these two polymorphisms is thought to increase the signs and severity of the abnormalities (15,18).

This study was conducted with the aim of investigating mutations of the MTHFR gene (C677T and A1298C) in patients with cleft lip and cleft palate by applying exact molecular methods and constructing a DNA bank of genetic information. The main objectives of the study were to increase the prevalence of the disease, cut the high cost of screening for abnormalities, generate epidemiological data, and collect data on risk factors such as family history, maternal age during pregnancy, parental occupation, number of siblings, and educational status of the family members. 

## Materials and Methods

*Study population*

This descriptive cross-sectional study was conducted at Tabriz Genetic Analysis Center, Tabriz University of Medical Sciences, Iran between 2010 and 2011. The study population was 65 affected children with non-syndromic cleft lips and cleft palates, and 130 of their parents. The diagnosis and type of cleft lip and cleft palate was confirmed by detailed physical examination during the child’s visit to the Ear, Nose, and Throat (ENT) clinic at Tabriz Children's Hospital (major child patient referral center for the North-West of Iran). According to the extent of their malfo- rmation, children were categorized as having cleft lip or cleft palate. 

Moreover, 50 healthy individuals with no background of cleft palate were recruited as the control group.

All parents and participants within the control group provided informed written consent, and the study protocol was approved by the Ethics Committee of Tabriz University of Medical Sciences (TUMS) in compliance with the Helsinki declaration.

*DNA Extraction*

Venous blood (5 ml) was extracted from all participants into coated ethylenedia- minetetraacetic acid (EDTA) test tubes. DNA was immediately extracted from the blood white cells using the phenyl-chloroform extraction method. As the final step, the quality and quantity of DNA extracted was examined. Two methods were used to assess the quality and quantity of genomic DNA: gel electrophoresis and ultraviolet (UV) spectrophotometry. 

*RFLP-PCR Performance*

In order to investigate the A1298C mutation of the MTHFR gene, the restriction fragment length polymorphism–polymerase chain reaction (RFLP–PCR) method was used. In accordance with the special primers (Table. 1), the relevant parts of genomic DNA were amplified and PCR reacted. The process was as follows: primary denaturizing level (95° degrees for 5 min); 35 cycles included denaturizing level (94° for 45 s); annealing level (62° for 45 s); extension level (72° degrees for 1 min); and the ultimate extension level (72° for 5 min). All these products were placed under electrophoresis by agarose gel (1.5%) and colored by ethidium bromide, respectively. 

**Table 1:** Primers and enzymes used in the RFLP_PCR process to investigate the A1298C mutation for MTHFR gene

**Mutation**	**Sequence of primers**	**PCR** **product (bp)**	**Restriction enzyme**	**Wild type**	**Heterozygote**	**Mutant**
MTHFR A1298C	F-CTTTGGGGAGCTGAAGGACTACTAC R-CACTTTGTGACCATTCCGGTTTG	163	MboII	56/31/30/28/18	84/56/31/30/28/18	84/31/30/18

*ARMS_PCR Performance*

To analyze the C677T mutation, the researchers used amplification refractory mutation system–polymerase chain reaction (ARMS-PCR). After proliferating the specified regions with special primers, the PCR reaction was undertaken under the following regimen: primary denaturizing level (96°for 2 min), 10 cycles including denaturizing level (96° for 15 s), 20 cycles including denaturizing level (96° for 10 s), annealing level (61° for 50 s), and extension level (72° for 30 s) (Table. 2). All products were placed under electrophoresis with agarose gel (1.5%) and colored with ethidium bromide, respectively.

**Table 2:** Special primers used in ARMS_PCR to investigate the C677T mutation of the MTHFR gene

Forward primer	5-TGC TGT TGG AAG GTG CAA GAT-3
Reverse primers	RW 5- GCG TGA TGA TGA AAT CGG-3
	RM 5- GCG TGA TGA TGA AAT CGA-3

It is worth mentioning that some genotypes of the mutation cases naturally occur (AA in A1298C, CC in C677T) in heterozygotes (AC about A1298C, CT about C677T) and homozygotes (CC in A1298C and TT in a C677T). In this study, both possibilities were studied. 

*Statistical Analysis*

Statistics were obtained and tabulated using SPSS 15. The findings are presented as average points, standard deviations, frequencies and percentage figures. Correlation between qualitative variables was presented through contingency tables after conducting the chi-square and Fishers Exact Test at the level of p<0.05 significance. 

## Results

A total of 65 affected children, 65 fathers, and 65 mothers, as well as 50 healthy individuals was selected as the sample in the present study. 

The age average was 1.4 years for the studied children, 30.2 years for the parents, and 32.8 years for the healthy individuals in the control group. A total of 44.6% of the study population was male and 55.3% female, while 21.5% of the patient children had a positive family history of abnormalities. 

Moreover, 38.5% of patients were exposed to one or more environmental risk factors such as smoking, drug addiction, antibiotics and corticosteroids, psychological and mental disorders, radiotherapy, and malnutrition (Table. 3). 

**Table 3:** Risk factors in cleft lips and cleft palates patients

**Gender**	**Male**	**Female**		
	29	36		
Family Disease history	No	Yes		
	51	14		
Complete pregnancy period	No	Yes		
	8	54		
Exposure to environmental risk factors	No	Yes		
	40	25		
Mother’s Age during Pregnancy	≥30	25-30	≤25	
	13	20	32	
Parents’ education level	HSD*≤E	HSD	E≤HSD	
	24	46	55	
Which child?	First	Second	Third or more	
	27	27	11	

* HSD=High school Diploma

The prevalence rates of the polymer- phisms C677T and A1298C for MTHFR gene in patients with cleft lips and cleft palates are shown in (Tables. 4,5).

**Table 4:** Genotype loci of the C677T mutation for MTHFR gene and the odds ratios

			**Genotypes**						**Frequency**		
Subjects	N		CC		CT		TT		677C	677T	Odds Ratio
			Nr	%	Nr	%	Nr	%	Nr	Nr	
Control G.	50		27	54	22	44	1	2	0.52	0.48	Ref.
CL/P Patients	65		38	58	25	38	2	3	0.55	0.45	0.44
CL/P's Fathers	67		37	55	27	40	3	4	0.51	0.49	0.64
CL/P's Mothers	67		33	49	33	49	1	1	0.48	0.52	1.35

As shown in Table 4, the C677T mutation for the MTHFR gene in cleft lips/palate patients (CL/P) was significantly different when compared with the control group (odds ratio [OR]=0.44). Moreover, the C677T mutation of the MTHFR gene in the CL/P mothers showed no significant difference compared with the control group (OR=1.35). However, the difference was significant compared with the mutation rates for the CL/P fathers and the control group (OR=0.64).

**Table 5:** Genotype loci of the A1298C mutation for MTHFR gene and the odds ratios

		**Genotypes**						**Frequency**		
Subjects	N	AA		CA		CC		1298A	1298C	Odds Ratio
		Nr	%	Nr	%	Nr	%	Nr	Nr	
Controls	50	13	26	34	68	3	6	0.2	0.8	Ref.
CL/P Patients	65	22	34	30	46	13	20	0.14	0.86	0.55
CL/P's Fathers	67	24	36	36	54	7	10	0.25	0.75	0.26
CL/P's Mothers	67	22	33	39	58	6	9	0.24	0.76	0.65
										

**Fig 1:**
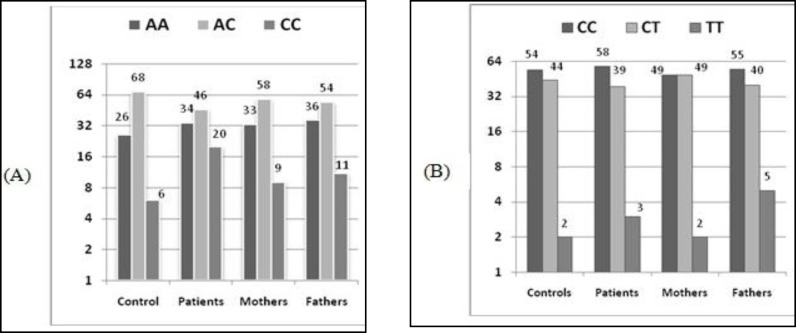
Frequency of natural types, homozygote and heterozygote loci of the C677T and A1298C mutations for MTHFR gene in CL/P patients, CL/P parents and control group

As illustrated in Fig.1A, the polymorphism C677T mutation for the MTHFR gene shows no significant difference between the CL/P patients, their fathers, mothers and control group in terms of naturalness (CC). However, this difference was significant in relation to the A1298C mutation (Fig. 1B). 

## Discussion

The results of the study reveal that the presence of the C677T mutation for the MTHFR gene in CL/P children and their fathers can be considered a risk factor in the development of cleft lips and cleft palates abnormalities. Several studies, however, have previously disapproved any strong associations between the C677T polymorphism and non-syndromic cleft lips and cleft palates (19-23). For instance, Verkleij-Hagoort et al. conducted 10 studies and showed no meaningful associations between cleft palates and the C677T mutation. This association was 0.5 in terms of mothers and 1 in patients (children) (24). However, in a study in Argentina, the rate of affected children who were homozygote to the C677T mutation was three times greater than in the control group (25). In another study in Ireland, an even stronger association was reported (26). 

Contrary to the findings of the current study, in two studies conducted in Italy, a strong association existed between the C677T mutation for the MTHFR gene, in CL/P patients’ mothers and the control group (21,27) that was in concordance with other studies (28,29). However, Jugessur et al. found a weak association between these mutations in CL/P patients’ mothers (30), whilst no association was seen in the study reported by Blanton (31).

The current study showed a significant association between the A1298C polymorphism of the MTHFR gene and non-syndromic cleft palates. This association was seen more strongly in CL/P patients’ fathers. This study more strongly supports an increased association in CL/P patients’ fathers compared with mothers. Few of the previous studies were able to prove an association between the mentioned polymorphism and the development of the CL/P abnormalities (15,23,31). Van der put and Friso reported that the A1298C polymorphism had no or little link to the development of the disorder (32,33). Their finding was supported by Pezzetti et al. who showed no direct association between this mutation and development of cleft lip and cleft palate abnormalities. They acknowledged the mutation as a potential secondary cause (27). 

Based on the findings in the present study, it can be understood that the relationship between the A1298C polymorphism with non-syndromic cleft palates, in comparison to the C677T mutation, is stronger in patients and their parents with respect to the control group. To date, similar findings have not been reported by other researchers. Other studies carried out in various countries suggest a much greater impact of the C677T polymorphism on the development of the cleft abnormalities. Similar studies are yet to be carried out in Iran; therefore, racial differences could be suggested as a reason for these novel findings. 

Accordingly, it is suggested that extensive and further studies should be carried out in the Middle East, as most current studies were conducted in European countries and in predominantly white populations. This study attempted to investigate the C677T and A1289C mutations as risk factors for the development of non-syndromic cleft abnormalities and can therefore be used as a platform for future studies. Finally, it can now been seen that more comprehensive and further studies on related genes are extremely important. 

## Conclusion

The presence of the C677T mutation for the MTHFR gene in CL/P children and their fathers can be considered a risk factor in the development of cleft lips and cleft palates abnormalities. The current study showed a significant association between the A1298C polymorphism of the MTHFR gene and non-syndromic cleft palates. This association was seen more strongly in CL/P patients’ fathers. This study more strongly supports an increased association in CL/P patients’ fathers compared with mothers. Based on the findings in the present study, it can be understood that the relationship between the A1298C polymorphism with non-syndromic cleft palates, in comparison to the C677T mutation, is stronger in patients and their parents with respect to the control group.
